# Finding mesopelagic prey in a changing Southern Ocean

**DOI:** 10.1038/s41598-019-55152-4

**Published:** 2019-12-12

**Authors:** Clive R. McMahon, Mark A. Hindell, Jean-Benoit Charrassin, Stuart Corney, Christophe Guinet, Robert Harcourt, Ian Jonsen, Rowan Trebilco, Guy Williams, Sophie Bestley

**Affiliations:** 1 Sydney Institute of Marine Science, 19 Chowder Bay Road, Mosman, New South Wales 2088 Australia; 2 Institute for Marine and Antarctic Studies, IMAS Waterfront Building, 20 Castray Esplanade, Battery Point, Tasmania 7004 Australia; 3 Department of Biological Sciences, Macquarie University, Sydney, New South Wales 2109 Australia; 4 Antarctic Climate & Ecosystems Cooperative Research Centre, University of Tasmania, Private Bag 80, Hobart, 7001 Australia; 5 L’Ocean, Université Pierre et Marie Curie, 4 Place Jussieu, 75005 Paris, France; 6 Centre d’Etudes Biologiques de Chizé, UMR 7372 Université de la Rochelle-CNRS, Carrefour de la, Canauderie, 79360 Villiers en Bois, France; 7 Present Address: CSIRO Oceans and Atmosphere, Hobart, Tasmania Australia

**Keywords:** Ecology, Climate sciences

## Abstract

Mesopelagic fish and squid occupy ocean depths extending below the photic zone and their vertical migrations represent a massive pathway moving energy and carbon through the water column. Their spatio-temporal distribution is however, difficult to map across remote regions particularly the vast Southern Ocean. This represents a key gap in understanding biogeochemical processes, marine ecosystem structure, and how changing ocean conditions will affect marine predators, which depend upon mesopelagic prey. We infer mesopelagic prey vertical distribution and relative abundance in the Indian sector of the Southern Ocean (20° to 130°E) with a novel approach using predator-derived indices. Fourteen years of southern elephant seal tracking and dive data, from the open ocean between the Antarctic Polar Front and the southern Antarctic Circumpolar Current front, clearly show that the vertical distribution of mesopelagic prey is influenced by the physical hydrographic processes that structure their habitat. Mesopelagic prey have a more restricted vertical migration and higher relative abundance closer to the surface where Circumpolar Deep Water rises to shallower depths. Combining these observations with a future projection of Southern Ocean conditions we show that changes in the coupling of surface and deep waters will potentially redistribute mesopelagic prey. These changes are small overall, but show important spatial variability: prey will increase in relative abundance to the east of the Kerguelen Plateau but decrease to the west. The consequences for deep-diving specialists such as elephant seals and whales over this time scale will likely be minor, but the changes in mesoscale vertical energy flow have implications for predators that forage within the mesopelagic zone as well as the broader pelagic ecosystem.

## Introduction

Despite their importance in marine food webs, very little is known about the factors influencing the distribution and abundance of the mesopelagic biota (fish, squid and macrozooplankton) or how this will change in the future^1–3^. The mesopelagic zone (200–1000 m) constitutes approximately 20% of the global ocean volume and plays a crucial role in global biogeochemical cycling^4^. Recent estimates suggest that mesopelagic fishes are the most abundant vertebrates in the biosphere^5^, with estimates varying from 1 × 10^9^ tonnes to 7 × 10^10^ tonnes but, major gaps remain in our knowledge of their biology^6,7^. A globally important characteristic of mesopelagic animals is their mass diel migration, where the animals rise toward the surface at night to feed and then return to deeper waters during the day^8^. This is the largest natural daily migration in the World’s oceans and represents an important mechanism for moving carbon and energy through the water column^9,10^.

Mesopelagic fish and squid constitute one of the principal pathways through which energy from primary producers is made accessible to higher order predators. In the Southern Ocean, these include flying seabirds, penguins, seals and whales; many of which are of high conservation value. Yet the mesopelagic biota is one of the least investigated components of the open ocean ecosystem, largely because the mesopelagic zone remains extremely difficult to observe and sample^11,12^. There are relatively few trawl-based and acoustic studies on the spatial (horizontal and vertical) distribution of mesopelagic fish and squid in the Indian sector of the Southern Ocean (*e.g*.^13–15^). These demonstrate that regionally, mesopelagic prey concentrate in an acoustically dense, deep scattering layer during the day (approximately 400–600 m) with a proportion migrating towards the surface during the night.

Understanding how ocean dynamics structure the mesopelagic habitat is critical for determining physical influences on the distribution and abundance of mesopelagic biota, how these drivers (and dependencies) are likely to change in the future, and the implications this has for the ecosystem as a whole. The Southern Ocean is a key region influencing the Meridional Overturning Circulation (MOC^16^) and therefore regulating the oceanic biogeochemical cycles^17–19^. Deep waters formed in the North Atlantic spread south as Circumpolar Deep Water (CDW). This is then transported in the deep layers of the Antarctic Circumpolar Current (ACC) and ultimately upwells south of the Polar Front^20^. The large-scale shoaling of warm, saline CDW is important for Southern Ocean productivity as it replenishes macronutrients (e.g. nitrate and phosphate) as well as micronutrients (such as iron) into the surface waters. Recent work indicates that an intensification of the MOC over 1995–2011 is linked to strong westerly winds, and has driven enhanced upwelling at high latitudes and important variability in oceanic carbon uptake^16,18^. Climate-related changes into the future are likely to have profound effects on all components of marine ecosystems^21^. These large-scale changes may lead to increased dominance of mesopelagic prey in Southern Ocean food webs, potentially with a corresponding decreasing importance of krill, but these changes are likely to be spatially variable and species-specific^22^.

A well-established approach to obtaining oceanographic data in remote realms is to attach state-of-the-art sensors to seals^23–25^. Biotelemetry instruments collect hydrographic data (temperature and salinity) concurrently with behavioural data (*e.g*. dive depth and duration) from the seal, providing important indicators of where prey are located in the water column^26^. As a biological observation platform^27^ elephant seals are a valuable component of a broader international observing system^25^. This system includes the Southern Ocean Carbon and Climate Observations and Modelling project^28^ and ship-based acoustic sampling^12^, providing indispensable observations for understanding biophysical processes in the ocean. Here, we harness these data in a new capacity for measuring and monitoring mesopelagic prey in time and space.

We compile oceanographic and behavioural data collected between 2004 and 2016 from adult female southern elephant seals (*Mirounga leonina*) foraging in the open ocean of the southern Indian Ocean (the region between the Antarctic Polar Front [APF] and the southern ACC front [SACCF]). These are combined with oceanographic conductivity, temperature and depth (CTD) data collected using Argo floats^29^ over the same period. We show that seal diving behaviour provides new information on the poorly understood distribution and dynamics of their mesopelagic prey and how this relates to ocean structure. We find that the vertical distribution of mesopelagic prey (inferred from predator dive depth) and their relative abundance (inferred from predator hunting indices) varies geographically with the relative depth of CDW. We use statistical models combined with projections of future oceanographic conditions to predict changes in mesopelagic prey distribution and relative abundance by 2100. Although expected changes are small overall there is notable geographic redistribution, with increasing relative abundances and shallower distributions predicted to the east of the Kerguelen Plateau and vice-versa in the west.

## Seals Reveal the Distribution and Abundance of Mesopelagic Prey in the Southern Ocean

**Figure 1:**
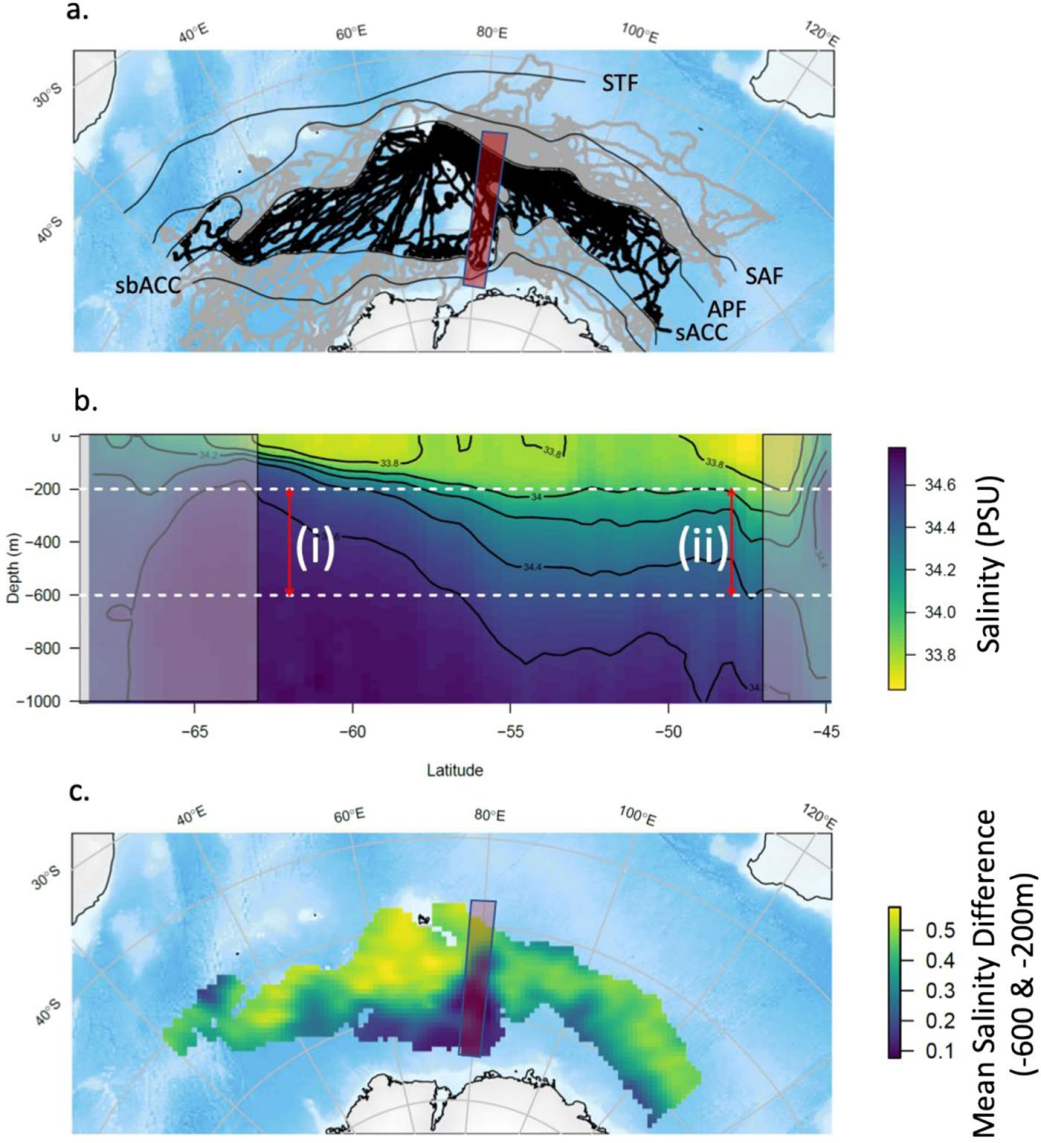
Spatial and vertical representations of the study domain. (**a**) Tracks from 98 adult females instrumented between 2004 and 2016. Tracks in the focal region of this study (the open ocean region of the Antarctic Zone) are highlighted in black and the grey are locations outside the study area. The solid black lines represent the climatological position of the major Southern Ocean fronts^20^. STF = subtropical Front, SAF = SubAntarctic Front, APF = Antarctic Poloar Front, sACC = southern Antarctic Circumpolar Current Front and sbACC = southern boundary of the Antarctic Circumpolar Current. The red box indicates the transect illustrated in (**b**). (**b**) A meridional transect centred along 80°E (5° degree width) showing the salinity of the upper water column from the surface to 1000 m. The relationship between S_diff_ (calculated as salinity at 600 m minus salinity at 200 m, dashed lines) and CDW is illustrated. Where CDW is shoaling towards Antarctica S_diff_ is small (shoaling CDW(i)). In comparison, farther north where CDW lies deeper in the water column S_diff_ is large (deep CDW(ii)). The shaded regions are outside our study domain. (**c**) A climatology of the salinity difference between 600 and 200 m (S_diff_) compiled from 14 years of seal and Argo float CTD data within the study domain between the Antarctic Polar Front and the Southern ACC Front.

In total, 98 adult female southern elephant seals were instrumented in the Indian sector of the Southern Ocean between 2004 and 2016 (Fig. 1a). We focus on female seals only because males tend to be continental shelf specialists that eat a high proportion of benthic prey^30,31^. We calculated the number of pelagic foraging dives made by the seals in each of the major oceanographic zones (Supplementary Material S1), demarcated using climatological positions of Southern Ocean fronts^20^. The Antarctic Zone (*i.e*., between the APF and the SACCF, excluding shelf areas) was used by 86 seals (88%) and more pelagic foraging dives were made in this zone than any other (n = 132,614; 39.3 ± 25.3% *per* seal). The next most important was the Subpolar zone (*i.e*., south of the Southern Boundary of the Antarctic Circumpolar Current: n = 62,023; 18.4 ± 26.3% *per* seal). Numerous diet studies, using multiple approaches including fatty acids^32–34^, isotopes^35–37^ and stomach contents^38–40^, have demonstrated that pelagically foraging elephant seals feed predominantly on fish and squid, hereafter referred to as “mesopelagic prey”, in the open ocean.

**Figure 2:**
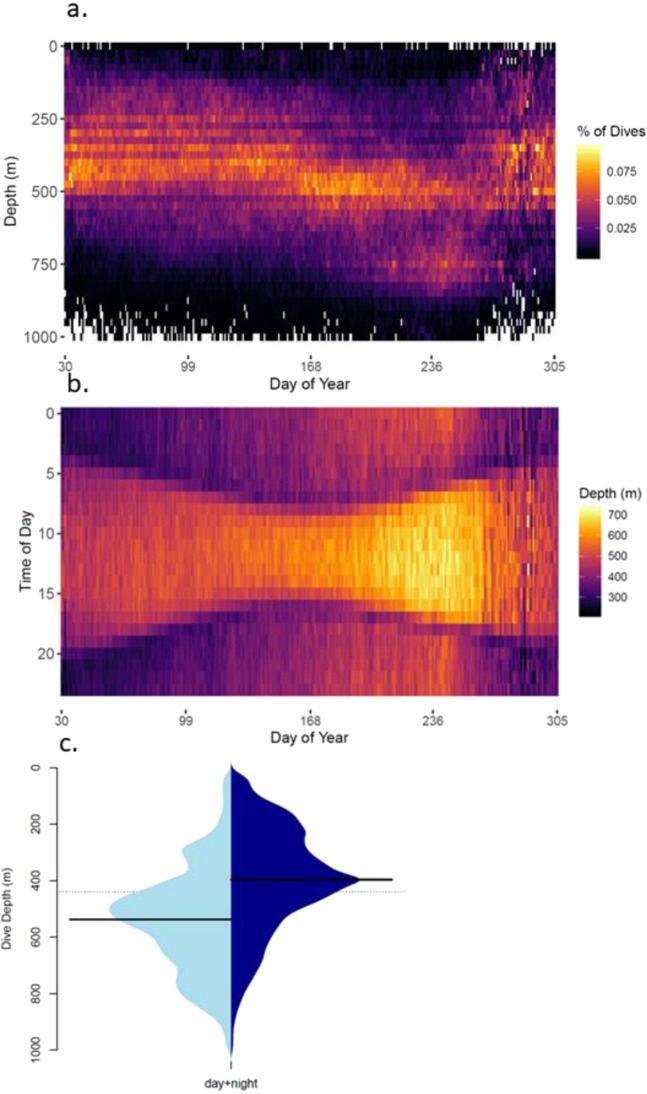
Aspects of female elephant seal diving behaviour at a range of temporal scales that indicate that they forage on mesopelagic prey. (**a**) The proportion of daily dives at each depth for each day during the post-moult period (February–October), (**b**) mean dive depth relative to the time of day and day of the year and (**c**) the distribution of dives depth during the day (light blue) and night (dark blue), with solid black lines indicting the mean depths.

The vertical distribution of seal pelagic foraging dives in time and space are consistent with what is known of the mesopelagic prey in this region^41^. Dive depths throughout the year bear a striking resemblance to the deep scattering layer of the mesopelagic zone^12^. The most common dive depths between 400 m and 600 m (Fig. 2a) coincide with the depth range of acoustically-detected myctophids with swim bladders^42^. The depth of pelagic foraging dives varies diurnally Fig. 2b), being on average 540 ± 178 m (mean ± SD across seals) during the day and 402 ± 182 m at night (Fig. 2c), consistent with the known daily migrations of dominant taxa in the mesopelagic zone; notably myctophids^8^. Moreover, this diel diving pattern evolves seasonally with the changing daylight hours (Fig. 2b), a known driver of fish vertical migration^8^. This is compelling evidence that the seals are foraging on mesopelagic prey as they move throughout their diurnal migration. Indeed, there is no other parsimonious explanation for the seals behaving in this way, given the comprehensive literature demonstrating that elephant seals are mesopelagic specialists^35,43–45^, and studies directly quantifying seal prey encounters show that mesopelagic prey are indeed encountered on the vast majority of dives^46–49^.

Further, it has been well demonstrated that the bottom time of a dive is positively related to the rate of prey encounters for elephant seals foraging in open ocean systems^26,50–52^. This has also been observed in a range of other diving predators^53–56^. More specifically, hunting time, as defined by reduced vertical movement rates, has recently been validated as the foraging index associated with the highest number of prey capture attempts for the types of summarised dive profiles we use in this study^57,58^. Taken altogether, this gives confidence that seal dive depth and hunting time provide effective indices of mesopelagic prey vertical distribution and their relative abundance.

### Influence of circumpolar deep water on mesopelagic prey

CDW is known as an important water mass for pelagically foraging elephant seals^59–61^. We use the difference in salinity between 600 and 200 m (S_diff_) as a metric to indicate the relative vertical position of saline CDW within the Antarctic Zone^62^. Where S_diff_ is large, CDW remains deep in the water column (Fig. 1b) underlying fresher surface waters. The spatial distribution of this metric (Fig. 1c) demonstrates the deeper distribution of CDW generally throughout the north of the Antarctic zone, with the strongest contrast between fresher surface waters and saline CDW evident immediately west of the northern Kerguelen plateau. We use *deep CDW* to describe high S_diff_ conditions, *i.e*., more differentiated near-surface and deep habitat for mesopelagic prey. Conversely, where S_diff_ is small CDW is relatively close to the surface, hereafter termed *shoaling CDW*. S_diff_ diminishes as CDW shoals southwards throughout the domain (Fig. 1c), with the northward intrusion of the western boundary current along the southern Kerguelen Plateau also apparent in the vicinity of 80°E.

**Figure 3:**
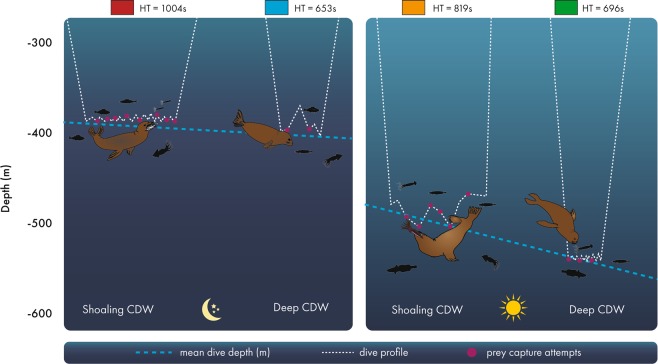
Conceptual representation of the relationship between seal  dive depth (m) and hunting time in relation to the time of day and the vertical proximity of Circumpolar Deep Water (CDW). The x-axis represents the difference in salinity from 600 m to 200 m (S_diff_), used as an index of the relative vertical position of saline CDW (S1c). Where S_diff_ is small, CDW is relatively close to the surface whereas larger values indicate CDW remains at depth. The y-axis indicates seal dive depth. The dashed blue lines are the fitted night time dive depths (left panel) from the model: depth ~ S_diff_ *day/night (S3), and the fitted day dive depths (right panel). The dashed white dive profiles are stylised seal dives. The coloured boxes  present a heatmap for the time seals actively hunted for prey, where red represents the longest hunting times (at night when CDW is shoaling), followed by day dives under shoaling CDW conditions (orange), day dives when CDW is deep (green) and night dives when CDW is deep (blue). The number of fish and squid in each dive pictorally represents a relative measure of feeding success following the above heatmap, showing that seal dives were most successful during night dives when CDW was shoaling, followed by day dives under shoaling CDW, day dives when CDW was deep, and least successful when diving at night when CDW was deep.

Linear mixed-models show the vertical distribution of the mesopelagic prey of elephant seals was directly related to S_diff_ (Supplementary Material S2), with the depth of pelagic foraging dives varying with both time of day and S_diff_. Modelled day time dives were shallowest (470 ± 15 m, mean ± s.e) in *shoaling CDW* conditions and deepest (547 ± 14 m) under *deep CDW* conditions. By contrast, night time foraging dives were relatively invariant, remaining around a depth of 400 m (range: 394 ± 14 m to 399 ± 14 m). This resulted in seals using a smaller range of the water column in regions where CDW was closer to the surface than where it was relatively deep (Fig. 3). Under *shoaling CDW* conditions the seals use on average 76 m of the water column (394 to 470 m) and under deep *CDW conditions* they use 148 m (399 m to 547 m). We infer that 400 m represents a depth of maximum night time prey opportunities; likely as it is the part of the water column where migratory members of the upper bathypelagic community co-occur with non-migratory mesopelagic prey^14,63^. This pattern is evident in sonograms^63^, and our results imply that this is relatively insensitive to the vertical position of CDW. In contrast, *shoaling CDW* appears to drive the vertical distribution of day time prey closer to the surface. Little is known about what drives the lower bound of the day time distribution of vertically migrating prey, but it is likely determined by an interplay of physical and biological processes. One possible explanation might be the trade-off between mesopelagic prey minimising their predation risk (under higher ambient light conditions), and maximising their forage opportunities *e.g*., where the permanent pycnocline (a density gradient established during deep winter mixing) aggregates biological particles as they rain down through the water column. High nutrient concentrations and/or oxygen minima associated with CDW may also be important in structuring mesopelagic prey distribution^44^.

The use of the water column along the gradient of S_diff_ (Fig. 3, Supplementary S2) suggests that mesopelagic prey may be more concentrated under *shoaling CDW* conditions (*i.e*. the prey are aggregated within a narrower part of the water column). This is supported by our finding that seal hunting time (see Methods) also relates to S_diff_. During the night, hunting time was 35% higher under *shoaling CDW* (1004 ± 44 s *per* dive) compared to d*eep CDW* (653 ± 44 s *per* dive) conditions (Fig. 3, Supplementary Material S2). We assessed model goodness-of-fit using conditional R-squared values^64^. This effect was similar, but less pronounced, during the day with an average difference of 15% between hunting times under *shoaling CDW* (819 ± 45 s) and d*eep CDW* (696 ± 45 s) conditions. Strong positive relationships between prey capture attempts and dive bottom time^52,65^, and hunting time^57,58,66^, have been demonstrated using head-mounted accelerometers^67^ on elephant seals foraging in this region. Accordingly, we infer that the relative density of mesopelagic prey is greater under *shoaling CDW* conditions, and highest during night time when prey vertical distribution is relatively shallow. This means that when the seals forage in regions of *shoaling CDW* prey encounter rates are considerably higher than when they must scan the broader water column in regions with *deep CDW*.

Another important determinant of the vertical distribution of mesopelagic fish is light intensity^68–71^. We have accounted for this to some extent by including day/night in our models to explicitly separate the diurnal migration of the mesopelagic prey. However, water transparency also has a well-known influence on distribution with fish moving deeper in the water column when light is more intense^68–71^. It is difficult to disentangle the effects of water mass properties and water transparency in the absence of data collected simultaneously on both. However, this problem relates mainly to the vertical distribution of the prey (*i.e*., one of our two metrics). Hunting time, our metric of relative mesopelagic prey density, is also related to CDW shoaling. As prey density *per se* is not known to be influenced by water transparency, this metric provides strong support for the influential role of oceanographic features. Further, shading of the water column from the presence of phytoplankton and zooplankton can directly affect water transparency^47,72,73^. Mesopelagic prey are also likely more abundant in shallower waters when there is higher productivity (i.e. more shading) in the upper water column. We therefore consider these elements to be internally consistent, and hypothesize that the primary mechanism for this productivity at the large spatial scale of our study is the relative proximity of the shoaling nutrient-rich CDW to the surface.

### Regionalised changes projected for 2100

We, like others^74^, compared current and future Southern Ocean conditions using the Max Planck Institute Earth System Model (MPI-ESM-MR) made available through the Coupled Model Inter-comparison Project, Phase 5 – CMIP5^75,76^. We calculated the mean salinities at relevant depths for the period 1970–1999 (from the historical simulation) to represent current conditions in the study domain. For future conditions we chose the RCP8.5 scenario as a representation of possible future conditions and averaged over the time period 2071–2100^77^. This represents the high end of emissions scenarios tested in CMIP5, where future CO_2_ concentration continues to rise at an increasing rate through to the end of this century. RCP8.5 was originally considered an extreme scenario, however for several years CO_2_ emissions continued to track this trajectory^78^. Using a lower emissions scenario will lead to smaller changes than those presented here. Using the relationships described in Fig. 2 and Supplementary Material S3, we estimate the change in mesopelagic prey vertical distribution and hunting time between the present and 2100 throughout the study domain within the southern Indian Ocean.

Projected changes in the future ocean show freshening of near surface waters in the west (30–70°E) versus increasing salinities in the east (70–130°E, see Supplementary Material S3, Fig. S3.1). Waters at depths near 600 m generally show increased salinities throughout the east (average increase of 0.0175 psu, Supplementary Material S3, Fig. S3.2). These changes are consistent with expectations of an intensification in the MOC (Conde Pardo *et al*., 2017 and references therein), driving both enhanced upwelling of saline CDW in the south and increased northward Ekman transport of fresh Antarctic Surface Water (AASW). This results in an overall change whereby S_diff_ becomes larger in the north and west and decreases in the east (Supplementary Material S3, Fig. S3.3).

**Figure 4:**
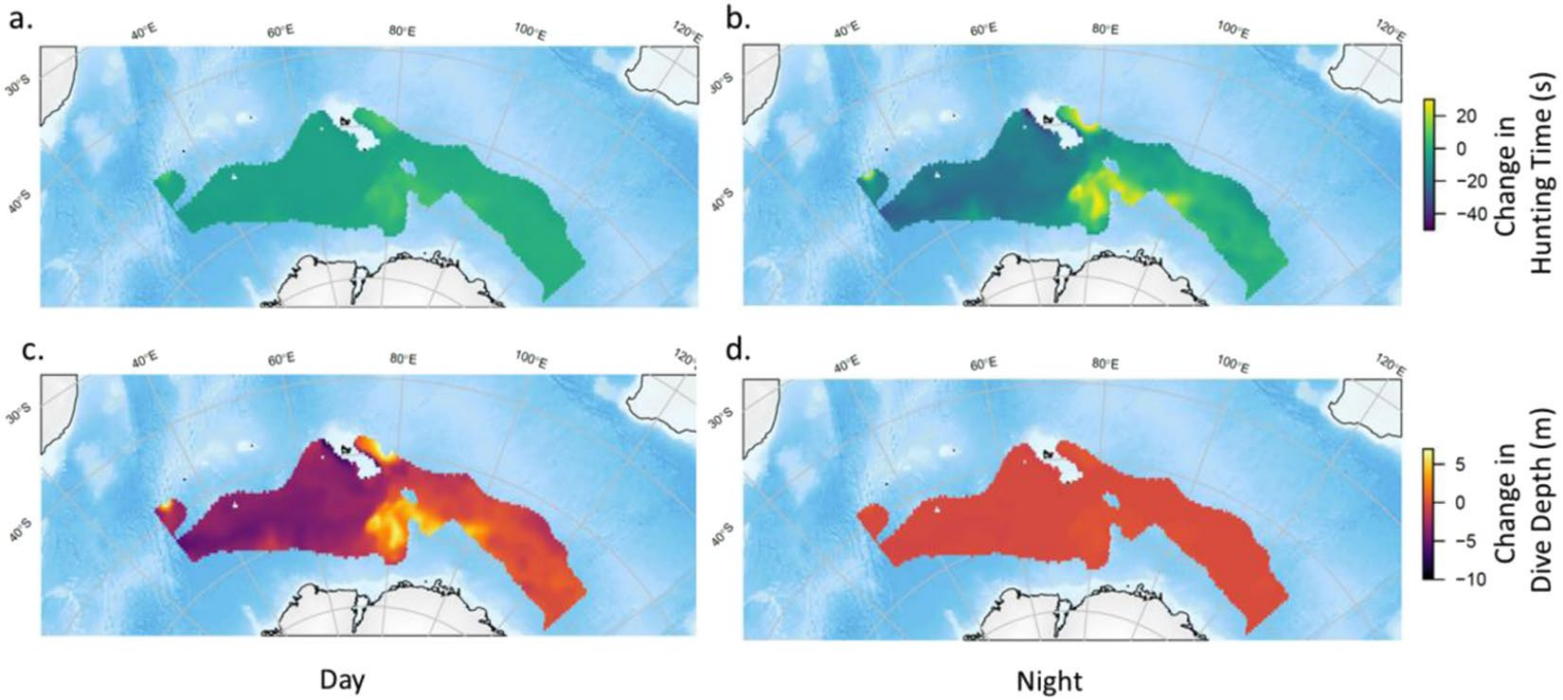
Projected change in vertical distribution and relative abundance of mesopelagic prey in the Antarctic Zone from the present to 2100. (**a**) hunting time changes (seconds) during the day and (**b**) hunting time changes during the night, showing that in the eastern part of the domain mesopelagic prey will be relatively more abundant (i.e. a positive change of up to 30 s hunting time), (**c**) dive depth changes (metres) during the day, showing that in the western part of the domain prey will be somewhat deeper (i.e. a negative change of up to 15 m), (**d**) dive depth changes during the night.

Despite the future changes in physical properties described above, overall the expected effect on mesopelagic distribution and relative abundance will be slight, albeit with some heightened geographic variation. The vertical distribution of mesopelagic biota (inferred from predator dive depth) will deepen in the western sector of the study region (Fig. 4) up to 15 m deeper in the west and 10 m shallower in the east. The relative abundance of mesopelagic prey (inferred from predator hunting time) decreases marginally in the west while increasing in the east; this change affects both day and night hunting but is most pronounced at night (Fig. 4, Supplementary Material S3). Again, these changes are relatively minor: up to 30 s more hunting time *per* dive in the east and only 5 s less in the west. Therefore, even under the most extreme climate change scenario, the biological implications for the energy pathway from mesopelagic prey through to higher trophic levels such and seals appear likely, at first glance, to be minor in this region. For elephant seals this translates to on average a one percent change in dive depths and only a 2% increase in terms of hunting time (Supplementary S4), which will be negligible in terms of individual foraging success and reproductive consequences^79^. However, smaller predators foraging on mesopelagic prey (e.g. Antarctic fur seals and king penguins) with lower aerobic capacity will likely be more substantially affected^80^.

Using seal dive metrics to study mesopelagic prey is a transformative innovation allowing us to assess and infer the spatio-temporal distribution of mesopelagic species in the Southern Ocean where in reality virtually nothing is known about broad scale patterns and drivers of abundance of this important group of animals^3^. This study has demonstrated that such an approach is complementary to other methods of studying mesopelagic fish and squid. Indeed, taking the cost, logistic uncertainty and restricted spatial and temporal scale of ship-based observations into account, predator behavioural data have a number of advantages including low cost and broad spatial and temporal coverage^24^. Rich opportunities exist for integrating predator data with the suite of tools for study mesopelagics, such as acoustic surveys^81^, increasingly acoustics on ships of opportunity, and large-scale ecosystem models^63^ to obtain a synoptic understanding of this enigmatic group of organisms.

## Materials and Methods

### Ethics statement

The study was carried out under approval from and in compliance of the the Australian Antarctic Science Program Animal Ethics for AAS project 4344.

### Seal telemetry data

We compiled 14 years of tracking data (2004–2016) from a total of 98 southern elephant seals deployed at two sites: Iles Kerguelen (49.35°S, 70.22°E) and Macquarie Island (54.50°S, 158.95°E). In all cases, a CTD-SRDL-9000 (Conductivity-Temperature-Depth Satellite Relay Data Logger – Sea Mammal Research Unit, St Andrews, UK) was used to provide 2–15 satellite location estimates each day, a random sample of individual dives, summarized into five time-depth segments and 2–4 CTD profiles each day^82^. To attach the instruments, the seals were chemically sedated^83^, weighed, and measured^84^, and the tag glued on the seal’s head^85,86^. The CTD-SRDLs remained on the seals until they either fell off or were shed during the annual moult.

At-sea seal locations were determined using the ARGOS satellite tracking system^82^, and then filtered using a state-space model with a 6-hour time step to estimate the most likely path for each individual, and its associated uncertainty^62^. We restricted our analyses to data from trips longer than 28 days to ensure we included some time at each seal’s foraging area, rather than simply the transit phase^87^. We also only included data from the seal’s winter post-moult trip (March to October). The final spatial domain of the tracking dataset extended from 20° to 130°E and from 35 to 80°S latitude, which encompassed 95% of all filtered locations.

### Study focal domain

This domain encompasses a great diversity of marine habitats including the Antarctic continental shelf, pack-ice, marginal ice zone, permanent open ocean zone and sub-Antarctic waters. These habitats are inhabited by diverse mesopelagic assemblages, notably nototheniid and myctophiid fish and numerous species of squid^14,88^. To reduce this complexity, in this study we focus on the Antarctic Zone south of the Antarctic Polar Front (APF) and north of the Southern ACC front (SACCF) based on climatological front position^20^.This region is in majority the permanently open oceanic zone. Here, the mesopelagic fish assemblage is dominated by lantern fish (*e.g*. *Electrona antarctica, Gymnoscopelus braueri, Krefftichythys andersoni* and *Protomyctophum tenisoni*). Important squid species in the area are thought to include *Histeoteuthis elantinae, Martialia hyadesi, Slosarczykovia circumantarctica, Galiteuthis glacialis*, *Mesonychoteuthis hamiltoni, Alluroteuthis antarcticus* and potentially *Moroteuthis knipovitchi*. However, the relative abundance of Southern Ocean squid is not well established due to their low catchability with commonly-used sampling equipment^88,89^.

We excluded dives made over the Kerguelen Plateau (defined here as waters shallower than 1000 m). We did this to account for any differences in faunal assemblages or behaviour over the shelf compared with the deep ocean. As prey may differ among elephant seal sex and age classes, we used only data from adult female seals, which feed predominantly on myctophids and squid when in open oceanic systems^32,35,37,90^. Each dive was then allocated to pelagic or benthic based on proximity to the ocean floor (using ETOPO1 bathymetry (www.ngdc.noaa.gov/mgg/global/): *Pelagic*, when the dive was at least 20 m above the ocean floor and *Benthic*, when the dive was within 20 m of the ocean floor. Benthic dives were excluded from statistical analyses. Finally, the years 2006 and 2008 were not included in analyses, due to few data in those years.

### Present oceanographic properties in the Antarctic Zone

We used two sources of ocean data: (i) Argo float conductivity-temperature-depth (CTD) profile data^29^ collected during March to October each year from 2004 to 2016. The Argo data were accessed from the Australian Ocean Data Network data portal (https://portal.aodn.org.au/AODN) and (ii) the CTD profiles collected by the elephant seals via the SRDL-CTD tags described above, downloaded from Marine Mammals Exploring the Oceans Pole to Pole - MEOP, (http://www.meop.net). All of these data were subject to post-hoc calibrations resulting in an accuracy of ±0.03 °C and to an accuracy better than ±0.05 for salinity^23^, sufficient to identify the major water masses of interest in this study^91^. For the seal CTD data, we estimated the location of each CTD using a linear interpolation of the filtered track based on the time of CTD cast.

We used the difference in salinity at depths between 600 and 200 m (S_diff_ = Salinity_600_ − Salinity_200_) for each CTD profile as a metric providing a measure of the relative position of saline Circumpolar Deep Water (CDW) in the water column. Where the difference is relatively large, CDW remains deep in the water column underlying fresher surface waters (*deep CDW*). Where the difference is small this indicates CDW is shoaling (*shoaling CDW*, *i.e*. rising nearer to the surface) southward towards Antarctica (Fig. 1). CDW is a water mass known to be important for foraging elephant seal^59,60,92^. Spatial variability in this metric across the study domain is presented by gridding the region into 100 × 100 km cells and calculating the mean S_diff_ of all the profiles in each cell for each year, and then aggregating these across all years.

### Mesopelagic prey distribution and relative abundance with respect to CDW position

A location for every dive was estimated using a linear interpolation of the filtered track based on the time of the dive. Two metrics were calculated for each dive: dive depth (the maximum depth of that dive, in metres) and the hunting time (an estimate of the time that seal was actively searching for prey, based on the vertical rate of change of a dive segment^58,93^. For all subsequent analyses, we restrict the data to dives that were most likely to be those in pursuit of mesopelagic prey defined here as *pelagic* dives, in the Antarctic Zone, with at least 60 s of hunting time.

We used linear mixed models to assess how dive depth and hunting time related to the oceanic variable S_diff_ (Supplementary Material S2). To account for diurnal patterns we also included *day/night* as a factor in the models, with individual seal (*ref*) nested within year as the random effect:

$$Depth \sim \beta \,y(i)+\beta ({S}_{diff})\ast day/night+\sigma \,y(i)\ldots Model\,1$$

$$Hunting\,time \sim \beta y(i)+\beta ({S}_{diff})\ast day/night+\sigma \,y(i)\ldots Model\,2$$

Examination of the model residuals indicated that the main effects did not require transformation, however the depth response variable was natural log transformed. We used Maximum Likelihood (ML) estimation when testing between models including each single variable and their interaction, and Restricted Maximum Likelihood (REML) for parameter estimation in the final model. Models were ranked using corrected Akaike Information Criterion (AICc) so that models with the lowest AICc received the highest ranking^94^.

### Future projections of mesopelagic prey in the Antarctic Zone

We chose to examine future conditions in the Southern Ocean using the Max Planck Institute Earth System Model (MPI-ESM,^75^. This model was part of the Coupled Model Intercomparison Project^76^ reported in the Fifth Assessment Report by the IPCC^95^. Recent work has discussed the importance of model selection for ecological applications^96^ and shown that models that have a reasonable representation of current conditions reduce uncertainty in projections of future responses. Cavanagh and co-authors showed that MPI-ESM was one the best models in the CMIP5 archive for reproducing current sea ice conditions in the Southern Ocean. Similarly, in a comparison of alternative models MPI-ESM was the most realistic describing Southern Ocean characteristics (mode water, mixed layer depth and stratification^97^. Specifically, here we used the mixed-resolution realisation (MPI-ESM-MR) that has increased horizontal resolution in the ocean, matching well with the scale of physical observations in the region, and with good performance representing the ACC^98^.

We compared model output from a historical run (1970–1999) to output from the RCP8.5 future scenario for the period 2071–2100^77^. This represents a high emissions scenario where future CO_2_ concentrations continue to rise at an increasing rate through to the end of the century. We averaged March-October spatial projections of salinity at 620 and 220 m to calculate S_diff.p_ for the present day and future periods (Supplementary Material S3). Then, we used Models 1 and 2 to predict dive depth and hunting time respectively across the study domain (for day and night time separately) to develop estimates for the position of mesopelagic prey in the water column and their relative abundance. Finally, we produced a map of the difference in depth and hunting time (future-present) across the study domain.

## Supplementary information

Supplementary information
