# Supplementary material for: Finding mesopelagic prey in a changing Southern Ocean
Source: Sci Rep. 2019 Dec 12;9:19013. doi: 10.1038/s41598-019-55152-4 (PMC6908696; doi:10.1038/s41598-019-55152-4)
Supplement: Supplementary file 1 — Supplementary information [file 41598_2019_55152_MOESM1_ESM.docx]

**Supplementary Materials**

**Finding mesopelagic prey in a changing Southern Ocean**

Clive R. McMahon ^1,2,3^*, Mark A. Hindell^2,4^*, Jean-Benoit Charrassin^5^, Stuart Corney^4^, Christophe Guinet^6^, Robert Harcourt^3^, Ian Jonsen^3^, Fabien Roquet^7,8^, Rowan Trebilco^2,4^, Guy Williams^2,4^ and Sophie Bestley^2,4^

^1^ Sydney Institute of Marine Science, 19 Chowder Bay Road, Mosman, New South Wales, 2088, Australia

^2^ Institute for Marine and Antarctic Studies, IMAS Waterfront Building, 20 Castray Esplanade, Battery Point, Tasmania, 7004, Australia

^3^ Department of Biological Sciences, Macquarie University, Sydney, New South Wales, 2109, Australia

^4^ Antarctic Climate & Ecosystems Cooperative Research Centre, University of Tasmania, Private Bag 80, Hobart 7001, Australia

^5^ L’Ocean, Université Pierre et Marie Curie

^6^ Centre d’Etudes Biologiques de Chizé, UMR 7372 Université de la Rochelle-CNRS, Carrefour de la, Canauderie, 79360 Villiers en Bois, France

^7^ Department of Meteorology (MISU), Stockholm University, Sweden

^8^ Department of Marine Sciences, University of Gothenburg, Sweden

*These authors contributed equally to this work

**Supplementary Material S1.** *Summary of the pelagic foraging dives made adult female southern elephant seals within the study domain between 2004 and 2016. The focal region for this study (APF-ACC) in indicated in bold.*

| **Ocean Zone** | **Number of seals** | **Total number of dives** | **Mean proportion of dives ± standard deviation** |
| --- | --- | --- | --- |
| sub-Tropical Front to sub-Antarctic Front (STF-SAF) | 19 | 17758 | 24.4 **±** 26.6 |
| sub-Antarctic Front to Antarctic Polar Front (SAF-APF) | 49 | 50470 | 32.9 **±** 25.7 |
| **Antarctic Polar Front to Antarctic Circumpolar Current (APF-ACC)** | **86** | **132614** | **39.3 ± 25.3** |
| Antarctic Circumpolar Current to Southern Antarctic Circumpolar Current (ACC-SACC) | 44 | 51292 | 23.3 ± 19.7 |
| Southern Boundary of the Antarctic Circumpolar Current (SACCs) | 33 | 62023 | 36.0 **±** 26.3 |
| Antarctic Shelf | 13 | 5602 | 16.1 **±** 26.8 |
| Kerguelen Shelf | 82 | 20136 | 23.4 **±** 36. |
| **Total** | **98** | **339895** |  |

**Supplementary Material S2.** *Results of  model 1, relating dive depth and salinity difference between 600 and 200 m (s_diff_), showing the fitted relationship between dive depth (m),  time of day (day/night) and s_diff_ . Also, the tresults of model 2 relating hunting time and salinity difference between 600 and 200 m (s_diff_), showing the fitted relationship between hunting time (s),  time of day (day/night) and s_diff_.*

***Table S2. Result of LMMs relating dive metrics and ocean properties.*** *Each model suite is ranked from the lowest AICc, and indicates (+) for factors and the coefficient for continuous variables which are included in each model. “diel” is a factor indicating if the dive was day/night. In both cases the random effect was random=~1|year/ref (see Methods).*

|  | **Intercept** | **diel** | **sdiff** | **diel\|sdiff** | **df** | **logLik** | **AICc** | **delta** | **Weight** | **Conditional**  **R^2^** |
| --- | --- | --- | --- | --- | --- | --- | --- | --- | --- | --- |
| **a. Dive Depth** | -469.7 | + | -96.75 | + | 7 | -427830 | 855674.1 | 0 | 1 | 0.396 |
|  | -495.1 | + | -36.42 |  | 6 | -427875.3 | 855762.6 | 88.52 | 0 |  |
|  | -511.1 | + |  |  | 5 | -427900.1 | 855810.3 | 136.19 | 0 |  |
|  | -412 |  | -73.51 |  | 5 | -431347.2 | 862704.3 | 7030.22 | 0 |  |
|  | -443.4 |  |  |  | 4 | -431431.8 | 862871.5 | 7197.42 | 0 |  |
| **b. Hunting Time** | 795.5 | + | 114.8 | + | -7 | 493447.9 9 | 86909.9 | 0 | 1 | 0.253 |
|  | 894.7 | + | 348.5 |  | -6 | 493513.6 9 | 87039.2 1 | 29.34 | 0 |  |
|  | 955.7 |  | 375.2 |  | -5 | 493705.6 9 | 87421.2 5 | 11.32 | 0 |  |
|  | 742.2 | + |  |  | -5 | 493715.2 9 | 87440.5 5 | 30.62 | 0 |  |

**Supplementary Material S3. Future projections.**

**Figure S3.1.** Salinity at 220 m (a) in the present, (b) in 2100, and (c) the difference between (a) and (b). The balck contours respesent the 1000 m isobaths.

**
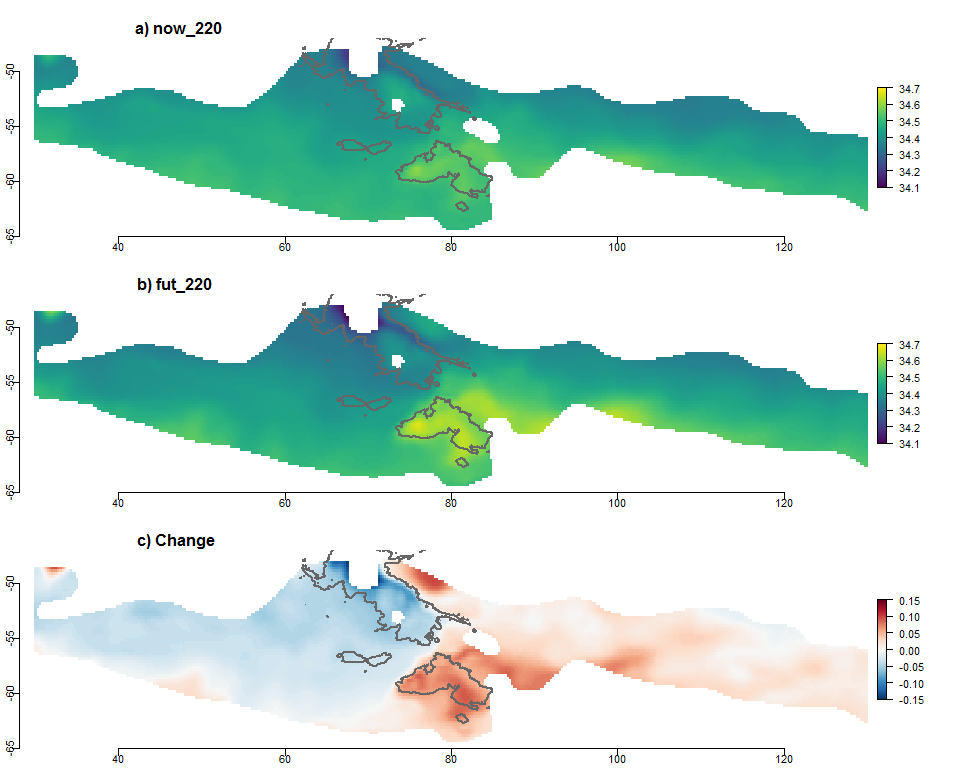
**

**Figure S3.2.** Salinty at 620 m (a) in the present, (b) in 2100, and (c) the difference between (a) and (b). The balck contours respesent the 1000 m isobaths.

**
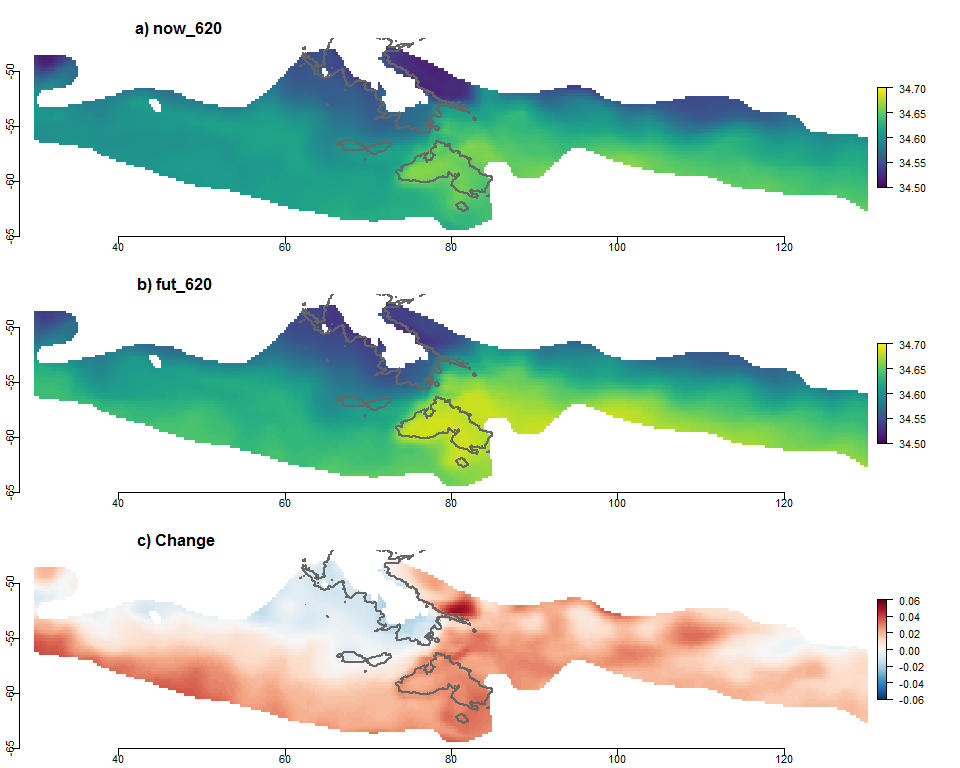
**

**Figure S3.3.** The S_diff_ metric (a) in the present, (b) in 2100 and (c) the difference between (a) and (b). The balck contours respesent the 1000 m isobaths.

**
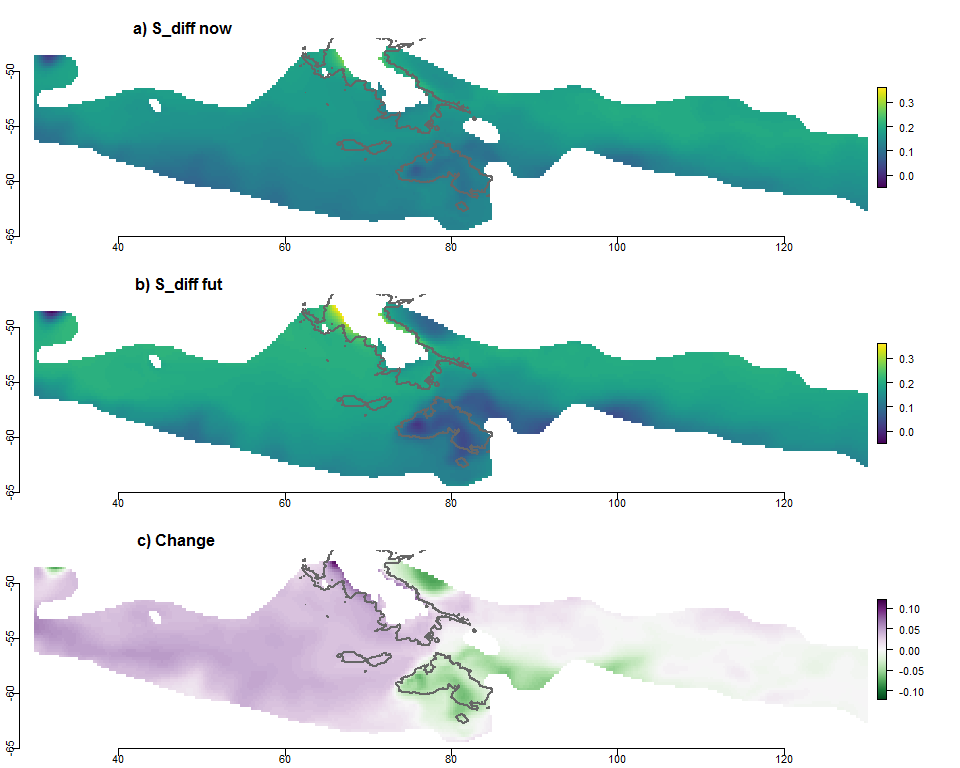
**

***Figure S4. The estimated degree of change in diving behaviour of southern elephant seals at Iles Kerguelen under CMIP5.***  *We calculated the dive depth and hunting time in each 10 x 10 km grid cell used by each of the 98 individual seals using predictions based on s_diff_ in that cell from models 1 and 2 respectively. From this, we calculated the overall mean depth and total hunting time for each seal. We did this for both current and future conditions and expressed the difference as percentage change from the present. We show the kernel density plots of this percentage for (a) dive depth and (b) hunting time. The shaded red curves represent day time and the blue night time.*

**
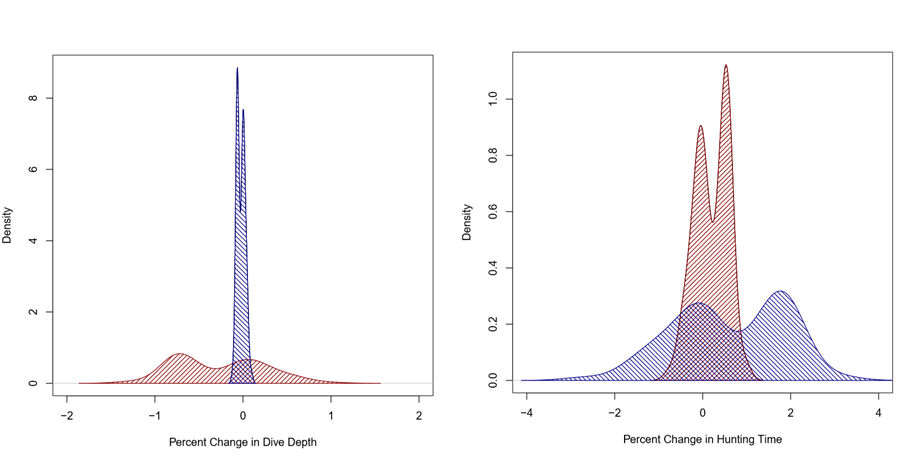
**
